# Relationship between academic procrastination and internet addiction in Peruvian university students: the mediating role of academic self-efficacy

**DOI:** 10.3389/fpsyg.2024.1454234

**Published:** 2025-01-23

**Authors:** Dana Rocio Chavez-Yacolca, Ruth Beatriz Castro-Champión, Nely Marlene Cisneros-Gonzales, Denis Frank Cunza-Aranzábal, Mardel Morales-García, Carlos D. Abanto-Ramírez

**Affiliations:** ^1^Unidad de Posgrado de Ciencias Humanas y Educación, Escuela de Posgrado, Universidad Peruana Unión, Lima, Peru; ^2^Facultad de Ciencias de la Salud, Universidad Peruana Unión, Tarapoto, Peru; ^3^Unidad de Salud, Escuela de Posgrado, Universidad Peruana Unión, Lima, Peru

**Keywords:** academic self-efficacy, internet, addiction, academic procrastination, university students

## Abstract

**Introduction:**

This study evaluated the mediating role of academic self-efficacy in the relationship between internet addiction and academic procrastination among Peruvian university students.

**Method:**

A cross-sectional explanatory design was employed with a convenience sample of 334 participants. Instruments used included the Specific Perceived Self-Efficacy Scale of Academic Situations (EAPESA), the Lima Internet Addiction Scale (EAIL), and the Academic Procrastination Scale (APS). The reliability and internal structure of each scale were verified, and the EAIL was validated. Descriptive analysis and correlation between variables were conducted, followed by path and mediation analyses.

**Results:**

Internet addiction significantly negatively affected academic self-efficacy (*β* = −0.381, *t* = −4.52, *p* < 0.001). Academic self-efficacy negatively impacted academic procrastination (*β* = −0.522, *t* = −7.04, *p* < 0.001). Internet addiction positively affected academic procrastination (*β* = 0.642, *t* = 5.72, *p* < 0.001). The total effect of internet addiction on academic procrastination and academic self-efficacy was significant (*β* = 0.841, *t* = 7.17, *p* < 0.001). Academic self-efficacy partially mediates the relationship between internet addiction and academic procrastination, suggesting universities should implement strategies to enhance academic self-efficacy in students.

## Introduction

Procrastination is a widespread failure in self-regulation that occurs when individuals delay or avoid starting essential tasks despite being aware of and wanting to do them (Senécal et al., 1995). This behavior manifests in several forms, including academic procrastination (AP), which may involve postponing essential academic activities until they become uncomfortable. This type of procrastination is particularly prevalent among college students and can significantly impact academic performance and mental well-being. Various studies indicate that PA is related to lower academic performance (Hayat et al., 2020; Okoye and Oghenekaro, 2020; Tian et al., 2021) and an increase in stress and anxiety levels (Ragusa et al., 2023), which significantly affects university academic life due to the accumulation of tasks and impending deadlines.

Recent research have identified certain factors that contribute to the academic performance of university students, among which intrinsic motivation can be noted. Various studies have demonstrated the efficacy of interventions focused on fostering enthusiasm and enjoyment among university students, such as personalized learning activities, collaborative learning through readings and videos, utilization of interactive elements as didactic resources, and implementation of augmented reality, among others (Alonso et al., 2023). Pedagogical approaches, such as flipped learning, have achieved significant improvements in critical thinking capacity and performance of university students (Atwa et al., 2022).

On the other hand, behavioral factors exist that negatively affect learning. A study conducted on university students in Spain found that those with suboptimal rest habits, specifically those who slept fewer hours, achieved lower academic performance.(Gallego-Gómez et al., 2021). Conversely, variables such as impaired mental health, manifested through anxiety, stress, or depression, negatively impact the learning and performance of university students (Aloufi et al., 2021). Furthermore, excessive academic demands in the university context produce professional burnout, which affects students’ academic self-concept and motivation to learn, ultimately impacting their performance (Wei et al., 2021). In light of this context of negative impact of stress on university students’ learning, self-efficacy constitutes a factor that can ameliorate this situation, empowering the student and propelling them towards academic success (Freire et al., 2020).

Additionally, Internet addiction (IA) has emerged as a contemporary challenge that has university students as a high-risk population (Sánchez-Fernández et al., 2023), and that intensifies procrastination. It is defined as excessive and problematic Internet use that is characterized by concerns, impulses, or uncontrolled behaviors related to Weinstein and Lejoyeux (2010). This addiction is associated with a greater inclination toward procrastination (Aznar-Díaz et al., 2020). It is distinguished from smartphone addiction, although both share similar characteristics, such as behavioral compulsions and a strong tendency to seek immediate gratification through technology (Panova and Carbonell, 2018). Furthermore, it is associated negatively with Academic self-efficacy (Li et al., 2020), suggesting procrastination may decrease belief in students’ ability to complete academic tasks successfully. Research indicates that the greatest challenges in internet management are found among university students, particularly first-year students, which may be related to a lack of self-control and reduced family supervision (Asrese and Muche, 2020; Ding et al., 2017) Although findings are not conclusive, male university students generally show a greater propensity for video games, while females tend to gravitate toward social media and online shopping (Baloğlu et al., 2020).

The relationship between academic procrastination and internet addiction is particularly notable, with studies documenting a clear link between high levels of internet addiction and a greater propensity to procrastinate in academic contexts (Abbasi and Izadpanah, 2018; Hayat et al., 2020; Zhang et al., 2022). In that context, self-regulation theory offers a useful perspective. It suggests that people may resort to compulsive Internet use to satisfy unmet desires or escape negative emotions and external pressures, leading to procrastination in academic or professional tasks (Chen and Lyu, 2024). This indirectly affects Academic self-efficacy, further deteriorating academic performance and time management among university students.

On the other hand, Academic self-efficacy (AA), which reflects students’ confidence in their ability to achieve success in academic tasks (Bandura, 1993; Palenzuela, 1983), has been identified as a crucial mediator in the relationship between Internet addiction and procrastination (Karakaya Özyer and Altınsoy, 2023). Greater self-efficacy may mitigate the effects of Internet addiction on procrastination, functioning as a buffer that reduces the tendency to procrastinate even in the presence of significant Internet addiction. This underscores the importance of strengthening Academic self-efficacy to combat both PA and the detrimental effects of AI, highlighting the role of self-regulated learning skills and perceived self-efficacy in academic performance.

In the Latin American context, certain characteristics of the educational model may influence this unhealthy practice, such as the predominance of rote memorization exams and the rigid pursuit of specific grades or honors as a synonym for academic achievement. Engineering and architecture faculties report the highest levels of procrastination. Among Peruvian university students, procrastination appears to be a cultural habit (Yarlequé Chocas et al., 2016). Variables such as work responsibilities (studying while working) and fear of failure increase the tendency to postpone academic tasks. Fear of failure contributes to disorganization and, consequently, poor time management, exacerbating procrastinatory behavior (Estrada Araoz and Mamani Uchasara, 2020; Manchado Porras and Hervías Ortega, 2021). A relationship has also been reported between internet or social media addiction and high levels of academic procrastination. Latin American university students tend to avoid any task perceived as difficult or boring. Spending excessive time online becomes a rewarding activity to avoid tasks, even those that are not inherently challenging or dull (George et al., 2023; Montag and Elhai, 2023). Procrastination negatively impacts academic self-efficacy, and women seem to regulate their activities better than men, although they are also more prone to anxiety related to academic procrastination (Altamirano Chérrez and Rodríguez Pérez, 2021; Burgos-Torre and Salas-Blas, 2020). Therefore, the objective of the present study is to explore the mediating role of Academic self-efficacy in the relationship between Internet addiction and academic procrastination in Peruvian university students.

## Method

### Design and participants

The research has a cross-sectional explanatory design and follows an associative strategy to establish the relationships between the variables, with an intervening variable acting as a mediator of said relationship (Ato et al., 2013).

For structural equation modeling, non-probabilistic convenience sampling was followed using the Soper calculator (Soper, 2024). This determined a minimum sample size of 269, an effect size of 0.5, a statistical power level of 0.8, and a significance of 0.05. However, a total of 334 Peruvian university students were considered. The ethical aspects of confidentiality of the data collected were considered, following the principles of the Declaration of Helsinki (Thatte et al., 2009).

### Instruments

The survey technique was used, for which the following scales were used:

To measure Academic self-efficacy, the Specific Perceived Self-Efficacy Scale of Academic Situations (EAPESA) was used. It was created in Spanish language by Palenzuela, 1983) and validated in Peruvian university students by Dominguez Lara (2018). It comprises 9 items and a single factor, with 4 response options (1 = never, 2 = sometimes, 3 = many times, and 4 = always). Evidence of validity concerning its internal structure (CFI = 0.978, GFI = 0.969, AGFI = 0.949, RMSEA = 0.056, SRMR = 0.029) and reliability (*ω* = 0.88) were reported for the Peruvian version.

Concerning Internet addiction, the Lima Internet Addiction Scale (EAIL) was used, developed in Spanish language, and validated in Peruvian adolescents by Lam-Figueroa et al. (2011). It consists of 11 items and two dimensions: symptomatologic characteristics (items 1–8) and dysfunctional characteristics (items 9–11), with 4 Likert-type response options (Very rarely = 1, rarely = 2, often = 3 and always = 4). The scale has proven reliable, with an *α* =0.84, and its factorial structure explained 50.7% of the total variance, demonstrating its factorial validity (KMO = 0.851).

To measure academic procrastination, the academic procrastination scale created by Busko (1998) was used, translated and validated in Spanish language by Álvarez Blas (2010), accordingly to the standardized processes of test translation and adaptation (International Test Commission, 2017), and validated in Peruvian university students by Dominguez-Lara et al. (2014) adhering to the required validity and reliability properties for translated instruments (American Educational Research Association, 2014), according to the purpose of the present study. The scale has 12 items and comprises two factors: Academic self-regulation (items 2, 5, 6, 7, 10, 11, 12, 13, and 14) and postponement of activities (items 1, 8, and 9). All items in the first factor are inverse. The scale has proven reliable (α = 0.816) and has adequate fit indices supporting its bifactor structure (CFI = 1.00, RMSEA = 0.078).

### Procedure

The research data were collected in accordance with the Helsinki Declaration guidelines, and approval was obtained from the research ethics committee of the Graduate School of Universidad Peruana Unión, which certified compliance with ethical standards and data protection (Reference: 2023-CE-EPG-00162). An online form was constructed using Google Forms, commencing with an informed consent request. This initial section informed participants about the altruistic and voluntary nature of their participation, the study’s objectives, procedures, and questionnaires to be completed. It emphasized the ethical use of data, confidentiality, anonymity, exclusive use of data for research purposes, the option to withdraw at any time, data protection, and access to results, all in compliance with necessary ethical research standards. Following the informed consent, the form included sections for sociodemographic data and data collection instruments. The researchers subsequently disseminated the form via WhatsApp and email to participants, requesting that each participant distribute the form link to their contacts who met the study’s required characteristics.

### Data analysis

Once the instruments were applied, the data were entered into the Jamovi 2.4.14 software, in which the descriptive analysis of the sociodemographic variables was carried out. Subsequently, the validity of the internal structure of the instruments was verified through confirmatory factor analysis using the SEMLj module (Gallucci and Jentschke, 2021). The items’ ordinal nature was considered, so the polychoric correlation matrix and the DWLS estimation method, which were considered the most appropriate for ordinal scales, were used (Finney and DiStefano, 2013). The fit evaluation was carried out through the comparative fit index (CFI), the Tuker-Lewis index (TLI), the root mean square error of approximation (RMSEA), and the standardized root mean square residual (SRMR). CFI and TLI values >0.90 and SRMR and RMSEA values <0.080 are considered appropriate (Kline, 2016; Mueller and Hancock, 2019).

The reliability of the instruments was analyzed using Cronbach’s alpha and McDonald’s Omega coefficients. Subsequently, the correlation between the variables under study was evaluated, and then the mediation hypothesis testing analysis was carried out.

After the validity and reliability analysis, descriptive statistics analysis was performed, including mean, standard deviation, skewness, and kurtosis. The mediation analysis was performed using the module medmod.

### Ethical considerations

It was approved by the ethics committee of the Universidad Peruana Unión (2023-CE-EPG-00162), respecting the ethical principles stipulated in the Declaration of Helsinki (World Medical Association, 2009).

## Results

Initially, the internal structure of the instruments was analyzed, obtaining appropriate adjustment values for the Scale of Perceived Self-Efficacy Specific to Academic Situations (EAPESA: χ^2^ = 13470.5, *p* < 0.001, CFI = 0.997, TLI = 0.996, RMSEA = 0.067 and SRMR = 0.043). For the Lima Internet Addiction Scale (EAIL), inadequate adjustment indices were initially obtained (*χ*^2^ = 7,596, p < 0.001, CFI = 0.974, TLI = 0.967, RMSEA = 0.123 and SRMR = 0.098), so four items were removed (2, 3, 4, 6), obtaining an adequate fit for a second model. Order (χ^2^ = 3709.3, *p* < 0.001, CFI = 0.996, TLI = 0.992, RMSEA = 0.067 and SRMR = 0.049). Regarding the Academic Procrastination Scale (APS), adequate fit indices were obtained for a second-order model (*χ*^2^ = 10,175, *p* < 0.001, CFI = 0.994, TLI = 0.992, RMSEA = 0.062 and SRMR = 0.054).

After confirming the evidence of validity of the internal structure of the instruments, their reliability was determined through the coefficients Cronbach’s (*α*) and McDonald’s (*ω*). As part of the descriptive analysis, the total scores of the scales were calculated, their average and standard deviation were determined, and the measures of skewness and kurtosis were found in a normal distribution range (±1.0). Then, the correlations between the variables under study were found (Table 1).

**Table 1:** Descriptive analysis, reliability of instruments, and correlation between variables.

	*M*	SD	g1	g2	α	*ω*	1	2
1. Academic self- efficacy (AS)	26.3	5.28	−0.014	−0.510	0.918	0.918	—	
2. Internet Addiction (IA)	10.7	3.49	0.772	0.351	0.848	0.857	−0.252***	—
3. Academic Procrastination (AP)	29.3	7.69	0.123	−0.160	0.866	0.872	–0.432***	0.380***

*p < 0.05, **p < 0.01, ***p < 0.001.

**Figure 1:**
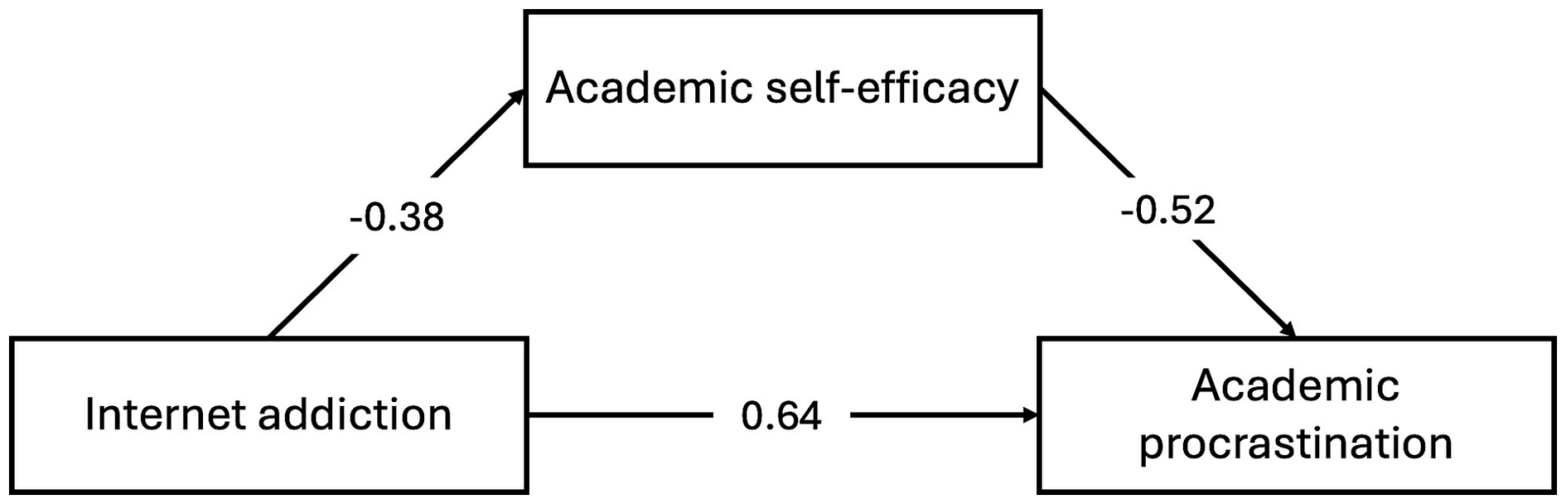
Statistical diagram.

Regarding the mediation analysis to evaluate the mediating role of Academic self-efficacy in the link between Internet addiction and academic procrastination, first, the effects between the variables were examined with path analysis, and the results are presented in Table 2 and Figure 1 where three different submodels were created. In Model 1, the effect of Internet addiction on Academic self-efficacy was measured. This effect was significantly negative (*β* = −0.381, *t* = −4.52, *p* < 0.001). Academic self-efficacy significantly negatively affected academic procrastination (Model 2) (*β* = −0.522, *p* < 0.001). The effect of Internet addiction on academic procrastination (Model 3) is positive and significant (*β* = 0.642, *p* < 0.001). When the bootstrap confidence interval is examined, it is concluded that all models are significant.

**Table 2:** Path analysis.

					95% Confidence interval			
			Estimate	SE	Lower	Upper	*Z*	*p*
IA	→	AS	−0.381	0.0843	−0.546	−0.216	−4.52	<0.001
AS	→	AP	−0.522	0.0742	−0.667	−0.377	−7.04	<0.001
IA	→	AP	0.642	0.1123	0.422	0.862	5.72	<0.001

IA, internet addiction; AS, academic self-efficacy; AP, academic procrastination.

**Figure 2:**
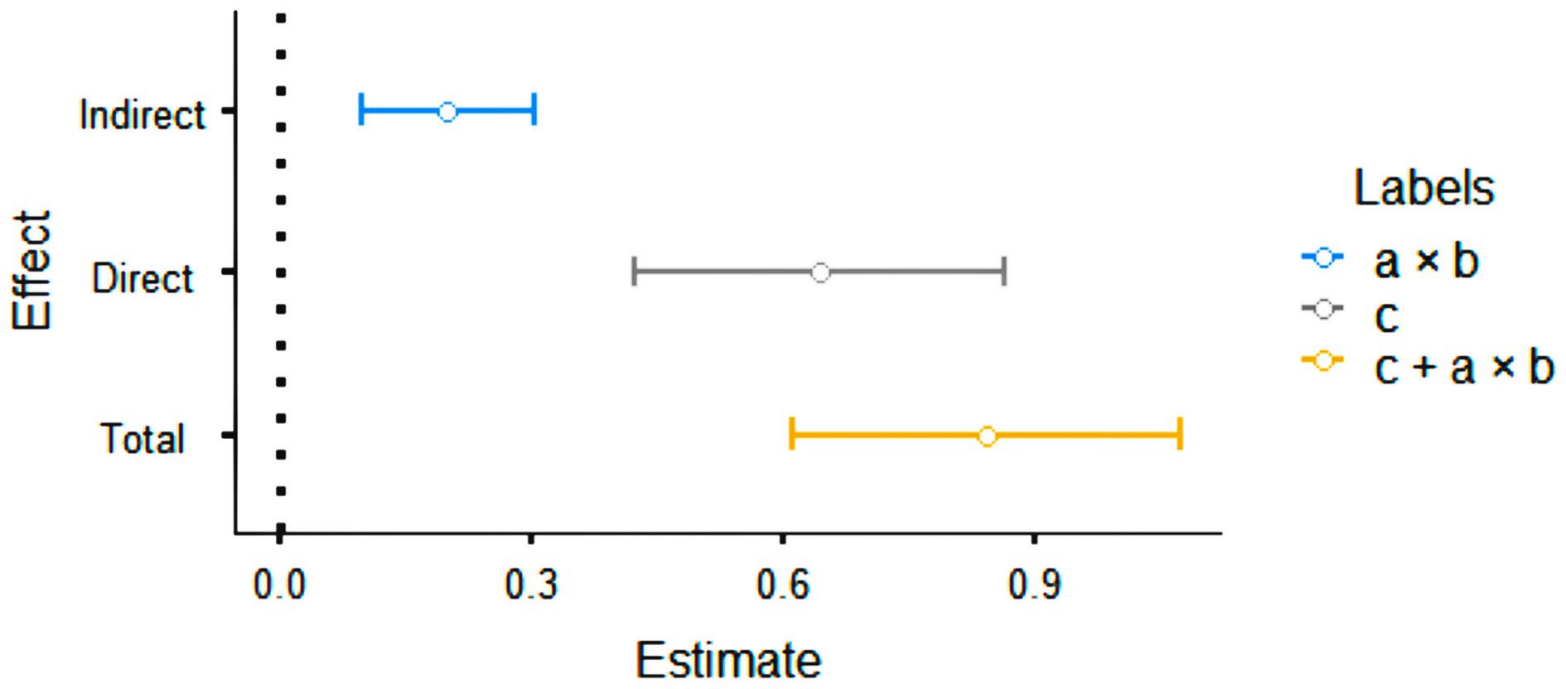
Graphic representation of the mediating role of Academic self-efficacy in the relationship between internet addiction and academic procrastination.

As shown in Table 3, the total effect of Internet addiction on academic procrastination and Academic self-efficacy was significant (*β* = 0.841, *p* < 0.001). With the inclusion of the mediating variable (Academic self-efficacy), the effect of internet addiction on academic procrastination maintained its significance (*β* = 0.642, *p* < 0.001). It was found that the indirect effect of Internet addiction on academic procrastination through Academic self-efficacy was significant (*β* = 0.199, *p* < 0.001). Therefore, the significant relationships necessary for the mediating effect have been identified. This shows that academic self-efficacy partially mediates, in a complementary way, the relationship between IA and AP (Zhao et al., 2010). It was determined that the percentage of mediation of Academic self-efficacy was 23.7%. However, the direct effect of Internet addiction on procrastination is 76.3%, which would indicate that Academic self-efficacy reduces the direct effect of Internet addiction on procrastination, although without achieving a total mediation effect. It is also possible to read the same result in the confidence interval of the bootstrap method. Consequently, given that the confidence intervals for the indirect effect and the total effect do not have a value of 0 (zero), the relationships here are significant (β = 0.199, SE = 0.0523, 95% CI = 0.0964; 0.301). The relevant data is shown graphically in Figure 2.

**Table 3:** Mediation estimates.

				95% Confidence Interval				
Effect	Label	Estimate	SE	Lower	Upper	*Z*	*p*	% Mediation
Indirect	a × b	0.199	0.0523	0.0964	0.301	3.80	< 0.001	23.7
Direct	c	0.642	0.1123	0.4218	0.862	5.72	< 0.001	76.3
Total	c + a × b	0.841	0.1172	0.6111	1.071	7.17	< 0.001	100.0

## Discussion

Internet addiction among university students increases their tendency to postpone completing academic tasks (Nadarajan et al., 2023). In this sense, it is also known that higher levels of self-efficacy are inversely related to academic procrastination and internet addiction (Karakaya Özyer and Altınsoy, 2023).

Therefore, this research aimed to explore the mediating effect of Academic self-efficacy in the relationship between internet addiction and procrastination in Peruvian university students.

Consequently, the mediation test showed that Academic self-efficacy was a complementary partial mediator in the relationship between Internet addiction and academic procrastination (Zhao et al., 2010). In other words, the effect of Internet addiction on academic procrastination occurs partially in a complementary way with Academic self-efficacy. This would indicate that Academic self-efficacy reduces the direct effect of Internet addiction on procrastination without exerting a total mediation effect. Furthermore, this would indicate that other variables not considered in the present study could contribute to the mediation between Internet addiction and academic procrastination.

As part of the three established models, Academic self-efficacy was found to have an inverse effect on internet addiction. The higher the levels of self-efficacy, the lower the incidence of internet addiction (−0.318). This coincides with what was reported by Yang (2020), although with a smaller effect size. Likewise, this is what was found by Agbaria and Bdier (2022), although it has a more significant effect size. Additionally, this study specifically focused on assessing Academic self-efficacy. This finding suggests that college students who feel confident and competent in their academic skills tend to use the Internet less addictively.

Likewise, it was determined that Academic self-efficacy has a negative effect on procrastination (−0.522). This implies that 27.2% of the variance in the decrease in academic procrastination is due to Academic self-efficacy. This finding corroborates what was found by Bozgün and Baytemir (2022) and Kurtovic et al. (2019), although the latter did not specifically assess Academic self-efficacy. This implies that self-efficacy has proven to be an important variable to consider if university students are to complete their academic tasks on time.

Regarding Internet addiction, it was found that it has a direct effect on academic procrastination (0.642). This means that about 41.4% of the variance in academic procrastination is due to Internet addiction; at higher levels of Internet addiction, university students tend to procrastinate with greater incidence. This result corroborates what was found by Can and Zeren (2019). Although unlike him, the target population was adolescents.

This study also demonstrated the psychometric properties of the Lima Internet Addiction Scale (EAIL) in university students. After applying the CFA, the two-dimensionality proposed by Lam-Figueroa et al. (2011), although 4 items were eliminated, resulting in a model with adequate fit indices (χ^2^ = 3709.3, *p* < 0.001, CFI = 0.996, TLI = 0.992, RMSEA = 0.067 and SRMR = 0.049). This is a significant contribution since it allows future researchers to have a valid and acceptable instrument to measure Internet addiction in Peruvian university students.

The present research has significant theoretical implications. It contributes to the understanding of temporal model of academic procrastination (Steel, 2007), by demonstrating that self-efficacy (expectancy, in Steel’s model) acts as a buffer against the effect of an impulsive factor, such as internet addiction, on academic procrastination. This suggests that addressing the issue of academic task non-compliance should focus not only on impulsive factors but also on strengthening students’ confidence in their abilities to achieve academic success, highlighting the importance of academic self-efficacy in the educational process and verifying its influence on an individual’s decisions, behavior, and attitudes, in this case, a university student, as proposed by social cognitive theory (Bandura, 1997). Thus, the significance of the self-regulation cycle in reducing academic procrastination, driven by self-efficacy, is emphasized (Zimmerman, 2000).

The present study has certain limitations. Causal relationships cannot be established since the data were collected at one point. It is also impossible to evaluate changes over time or trends regarding the behavior of the study variables. Furthermore, the sample was selected by convenience, so it may need to be more representative and may have caused bias in the results. It is recommended that future studies address longitudinal studies to establish causality between the variables addressed. Furthermore, it is recommended to investigate the relationship between these variables with larger samples and in diverse sociocultural contexts. Furthermore, it is recommended to conduct studies that incorporate additional factors into the model which, similar to internet addiction, are detrimental to university students’ learning processes. This approach would facilitate the implementation of curricular and didactic strategies to foster an enriching academic experience and to establish effective coping mechanisms for addressing adverse situations in this process.

In conclusion, Academic self-efficacy is a partial mediator of the relationship between Internet addiction and academic procrastination in Peruvian university students. According to the findings, higher education institutions are suggested to implement programs and strategies that strengthen university students’ self-confidence in their academic abilities, such as mentoring programs (Chelberg and Bosman, 2020), peer feedback (Fatima et al., 2022) etc., so that students complete their academic activities on time by reducing the effect that internet addiction has on their procrastination. Additionally, workshops focused on the development of emotional skills, such as self-regulation, should be implemented periodically to reduce internet addiction levels and its impact on procrastination (Meng and Zhang, 2023).

Furthermore, the application of gamification activities would be beneficial in redirecting internet usage towards gratifying learning activities, potentially reducing addiction levels (Li et al., 2021). It is imperative that educators raise awareness among their students regarding the significance of time management and recommend digital tools, such as RescueTime, Freedom, StayFocusd, among others, which enable them to regulate the time spent on non-academic activities on the internet, thereby reducing the propensity for procrastination (Deng et al., 2023). Los docentes deberían incorporar estrategias que fortalezcan la autoeficacia académica entre sus estudiantes. Por ejemplo, realizar tutorías para ayudarlos a establecer metas académicas claras, fortaleciendo su confianza para alcanzarlas.

In alignment with historical-cultural approach (Vygotsky, 1934), in addition to redirecting students’ internet usage from a distracting medium to a platform that provides tools contributing to the teaching-learning process, it is imperative to promote critical and reflective reading through online discussion forums and activities that foster the development of critical thinking and argumentation, with the aim of enhancing their capabilities and increasing their self-efficacy (Abanto-Ramirez et al., 2024; Zambrana Ortiz, 2016).

## Data Availability

The raw data supporting the conclusions of this article will be made available by the authors, without undue reservation.
